# Influence of Change in Aerobic Fitness and Weight on Prevalence of Metabolic Syndrome

**DOI:** 10.5888/pcd9.110171

**Published:** 2012-03-08

**Authors:** Laura A. Crist, Catherine M. Champagne, Leonor Corsino, Lillian F. Lien, Guangyu Zhang, Deborah Rohm Young

**Affiliations:** University of Maryland School of Public Health, College Park, Maryland; Pennington Biomedical Research Center, Louisiana State University System, Baton Rouge, Louisiana; Duke University Medical Center, Durham, North Carolina; Duke University Medical Center, Durham, North Carolina; University of Maryland School of Public Health, College Park, Maryland; Department of Research and Evaluation, Kaiser Permanente Southern California. At the time of the study, Dr Young was affiliated with the University of Maryland School of Public Health, College Park, Maryland

## Abstract

**Introduction:**

The metabolic syndrome is the clustering of several cardiometabolic risk factors that can lead to the development of coronary heart disease and type 2 diabetes. We evaluated whether a change in aerobic fitness resulting from a lifestyle intervention could significantly change the odds of metabolic syndrome prevalence.

**Methods:**

Participants (n = 810) were recruited into PREMIER, a multicenter, randomized, controlled clinical trial with outcome assessments at 6 and 18 months. The primary eligibility criterion was a diagnosis of prehypertension or stage 1 hypertension. PREMIER randomized participants to 2 lifestyle interventions, both of which included increased physical activity, or an advice-only control group. Participants completed a submaximal treadmill exercise test; we used reduction in heart rate as the measure of improved aerobic fitness. We used logistic regression to determine intervention effects on metabolic syndrome prevalence. Our models controlled for dietary pattern change.

**Results:**

The lifestyle interventions had no significant effect on metabolic syndrome prevalence at 6 months or 18 months. When combining intervention and control groups, at 6 and 18 months, a 1-beat-per-minute reduction in heart rate was associated with a 4% reduction in prevalence of metabolic syndrome (*P* < .001). When we tested for weight change as a mediator, the association was no longer significant.

**Conclusion:**

Increased aerobic fitness may reduce prevalence of metabolic syndrome. This association appears to be mediated through concomitant weight change.

## Introduction

Metabolic syndrome is the clustering of several cardiometabolic risk factors namely high waist circumference; high triglycerides, blood pressure, and blood glucose; and low high-density lipoprotein  and can lead to the development of coronary heart disease and diabetes (1). The prevalence of metabolic syndrome is high (2), and it is associated with morbidity and all-cause mortality (3-5). According to the 2003-2006 National Health and Nutrition Examination Survey, the US prevalence of metabolic syndrome is approximately 34% (6). Prevalence is particularly high among Hispanics and African Americans (6).

It is well documented in cross-sectional studies that physical activity (2,7,8) and in longitudinal studies that high levels of physical fitness are associated with lower prevalence of metabolic syndrome (9-13). To our knowledge, few longitudinal studies show the preventive effect of fitness on metabolic syndrome (1,3,14), and only 1 included substantial racial and ethnic diversity (11). These results suggest that an aerobic fitness intervention should have a protective effect for people without metabolic syndrome and a therapeutic effect for those with metabolic syndrome. Several intervention studies found that an increase in exercise can help some participants with metabolic syndrome improve their cardiometabolic risk factors so that they are no longer classified as having metabolic syndrome at follow-up (15-17). To our knowledge, only 2 randomized controlled trials testing this association have been published, and results are conflicting (17,18).

According to a review by Ford and Li (14), no randomized controlled trials with sufficient sample size and generalizable demographic characteristics have examined the effect of physical activity or physical fitness on metabolic syndrome. Therefore, we conducted a secondary analysis of PREMIER, a randomized, controlled trial that examined the effects of 2 lifestyle interventions on blood pressure (19). We hypothesized that participants in the lifestyle interventions would have lower prevalence of metabolic syndrome compared with participants in the control group. In post-hoc analyses, we 1) examined how change in fitness, irrespective of assignment to intervention or control group, was associated with change in metabolic syndrome, 2) evaluated the mediating effect of weight change on fitness change and metabolic syndrome prevalence, and 3) examined which metabolic syndrome components were associated with fitness change. One-third of the study participants were African American, providing racial diversity lacking in previous work.

## Methods

PREMIER was a randomized, controlled trial conducted from 1998 to 2004 at 4 clinical sites: Johns Hopkins University, Baltimore, Maryland; Pennington Biomedical Research Center, Baton Rouge, Louisiana; Duke University Medical Center, Durham, North Carolina; and Kaiser Permanente Center for Health Research, Portland, Oregon. Institutional review boards at each center approved the study, and all participants provided written informed consent. The main outcome results were published in 2003 (19).

### Participants

PREMIER staff recruited participants into the trial through mass media outlets. African Americans were overrecruited to obtain adequate African American representation (20). Major inclusion criteria were: at least 25 years old, diagnosed with prehypertension (systolic blood pressure, 120-139 mm Hg; diastolic blood pressure, 80-89 mm Hg) or stage 1 hypertension (systolic blood pressure, 140-159 mm Hg; diastolic blood pressure, 90-95 mm Hg), and a body mass index (BMI, in kg/m^2^) ranging from 18.5 to 45.0. Major exclusion criteria were taking blood pressure or weight-loss medications, medical history of cardiovascular disease or stroke, pregnancy, psychiatric disabilities, and major weight change in the 3 months before screening. Approximately 4,000 participants attended at least 1 screening visit. Of these, 3,154 were ineligible because of blood pressure being too low (n = 2,103) or too high (194) or because of other exclusions (n = 857), leaving 810 participants (19).

### Interventions

Staff randomly assigned participants to 1 of 3 groups: an advice-only control group (AO), a comprehensive lifestyle intervention group, termed the Established Group (EST), or a comprehensive lifestyle intervention plus Dietary Approaches to Stop Hypertension (DASH) diet group, EST+DASH.

Interventionists gave AO participants recommendations based on the National High Blood Pressure Education Program (21). The recommendations included weight loss if overweight, limiting alcohol and dietary sodium intake, regular physical activity, and eating a healthy diet. Interventionists distributed written materials at the baseline visit and again at the 6-month follow-up visit. They did not provide behavioral counseling.

The EST and the EST+DASH groups received a multicomponent lifestyle intervention, an 18-month program based on the most current clinical practice guidelines for blood pressure control and cardiovascular health. The guidelines were 1) weight loss of at least 15 lb (6.8 kg) at 6 months for those with a BMI of at least 25, 2) at least 180 minutes per week of moderate-intensity physical activity, 3) daily intake of no more than 100 mEq of dietary sodium, and 4) daily intake of 1 oz or less of alcohol. Interventionists instructed the EST+DASH group how to eat according to the DASH diet, which emphasized eating 9 to 12 servings of fruits and vegetables and 2 to 3 servings of low-fat dairy products per day while limiting fat intake to less than 25% of total calories and saturated fat to less than 10%. Although the dietary component for the 2 intervention groups differed, intervention goals and schedules regarding physical activity, sodium intake, and weight loss were identical. Because the intervention groups had the same physical activity goals, we combined them into a single group for the purposes of our analysis.

### Measures

Our main outcome was the prevalence of metabolic syndrome, as defined by the National Cholesterol Education Panel-Adult Treatment Panel III (NCEP-ATP III), at 6-month or 18-month follow-up (22). The NCEP-ATP III defines metabolic syndrome definition as the presence of 3 or more of the following risk factors: central waist circumference greater than 88 cm for women or 102 cm for men, triglycerides higher than 149 mg/dL, high-density lipoprotein (HDL) cholesterol less than 50 mg/dL for women or 40 mg/dL for men, blood pressure higher than 129 systolic or 84 diastolic or taking blood pressure-lowering medication, and fasting blood glucose greater than 109 mg/dL (23). Although taking blood pressure medicine was an exclusion criterion for our study, participants who remained hypertensive at 6-month follow-up were prescribed such medication.

Staff assessed aerobic fitness using a submaximal treadmill exercise test developed for PREMIER (24). We designed the test to achieve age- and sex-specific moderate intensity (approximately 60% of maximal metabolic equivalents [METS], in which 1 MET equals the resting metabolic rate). The protocol lasted 10 minutes, including warm-up and cool-down. We obtained a heart rate reading at the end of each minute. We considered the test complete when the participant reached 85% of his or her age-predicted maximal heart rate (220 minus age) or when the protocol was completed. Consistent with prior publications of PREMIER, we used heart rate at the end of stage 2, or the last available stage for participants who stopped early, for our analyses (19). Aerobic fitness change was defined by the difference in stage 2 heart rate from baseline to 6 months and baseline to 18 months. Heart rate is highly correlated (>.90) with oxygen uptake (25). A lower heart rate at a given workload implies a fitness improvement.

Trained staff took blood pressure measurements using a random zero sphygmomanometer (26) after the participant sat quietly for at least 5 minutes. We obtained 2 measurements at each visit using an appropriate-sized cuff. Four sets of blood pressure measurements were taken over 3 months and averaged to determine baseline blood pressure. Six sets of measurements over 3 visits were used to determine blood pressure at 6 and 18 months. Staff measured waist circumference using an anthropometric measuring tape.

Triglycerides and HDL cholesterol were measured from a blood sample obtained after a 12-hour fast. The Core Laboratory for Clinical Studies at Washington University, St. Louis, Missouri, performed the analyses. Serum blood glucose levels were measured using standard procedures.

### Analysis

We classified participants by metabolic syndrome status at baseline, 6 months, and 18 months. For participants with missing data for 1 or more of the metabolic syndrome criteria, we applied the following adjustments: if only 1 criterion was missing but the participant still had 3 or more risk factors, he or she was classified as having metabolic syndrome. However, if 2 or more criteria were missing and the participant did not have 3 risk factors, we eliminated the participant from the analysis to reduce misclassification (27).

We used logistic regression to calculate β coefficients and standard errors (SEs) for intervention effects on metabolic syndrome prevalence. In post-hoc analyses, we also used logistic regression to examine the effect of change in level of aerobic fitness on metabolic syndrome prevalence, regardless of intervention or control group status. Physical activity was the intervention modality to increase aerobic fitness in this study. However, we chose change in fitness as the independent variable because our previous results indicated intervention-related changes in fitness but not in physical activity (19).

Initial models suggested that weight change was a significant predictor of change in metabolic syndrome status, so we conducted additional analyses to determine whether potential associations between aerobic fitness and metabolic syndrome prevalence were independent of or mediated by weight change. Mediation was assessed using the procedures of Baron and Kenny (28). We used logistic regression models to calculate odds ratios (ORs) and confidence intervals (CIs) for the effects of aerobic fitness on cardiometabolic risk factors, both with and without including weight change. All models included the covariates race, sex, age, clinical center, cohort, baseline BMI, baseline aerobic fitness, baseline metabolic syndrome status, and dietary variables. We adjusted for the specific dietary components of daily calories, alcohol consumption, fruit and vegetable servings, low-fat dairy servings, percentage of kilocalories from fat and saturated fat (all assessed from three 24-hour dietary recalls and analyzed by Nutrition Data System NDS-R 1998 (University of Minnesota, Minneapolis, Minnesota), and sodium intake (assessed from 24-hour urinary secretion of sodium). Significance was set at *P* < .05. We used SAS version 9.1 (SAS Institute, Inc, Cary, North Carolina) for our analyses.

## Results

Of the 810 randomized participants, we excluded 6 because of missing data needed to classify metabolic syndrome. Complete follow-up data were available for 665 participants (82%) at 6 months and 656 participants (81%) at 18 months. The mean age in both groups was approximately 50 (Table 1). Overall, approximately two-thirds of participants were female and approximately one-third were African American. Baseline fitness levels were the same in both intervention and control groups. Approximately half of the participants had metabolic syndrome.

**Figure. F1:**
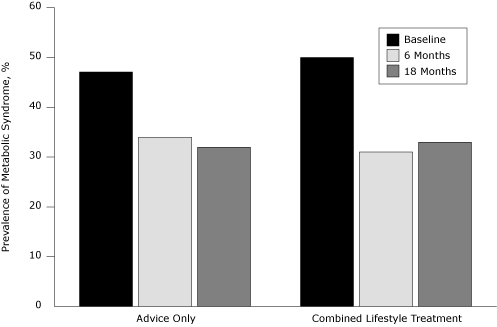
Prevalence of metabolic syndrome, by treatment status, at baseline and 6- and 18-month follow-up among participants in the PREMIER trial, Louisiana, Maryland, Oregon, North Carolina, 1998-2004.

Prevalence of metabolic syndrome decreased to 34% at 6 months and 32% at 18 months in the control group and to 31% at 6 months and 33% at 18 months in the combined lifestyle treatment (CLT) group ([Fig F1]Figure). Lower fitness was significantly associated with higher prevalence of metabolic syndrome at baseline, 6, and 18 months.

**Table:** 

Group Assignment	Prevalence of Metabolic Syndrome, %		

	Baseline	6 Months	18 Months
**Advice only**	47	34	32
**Combined lifestyle treatment**	50	31	33

At 6 months, the CLT group significantly increased aerobic fitness relative to the AO group (mean heart rate reduction [SE]: CLT, −8.52 [0.52] beats/min; AO, −5.31 [0.64] beats/min). Fitness was not significantly different at 18 months (mean heart rate reduction [SE]: CLT, -8.84 [0.52] beats/min; AO, −7.38 [0.68] beats/min). In our primary analysis, intervention or control group status had no effect on prevalence of metabolic syndrome at 6 or 18 months. Relative to AO, the CLT group had odds of metabolic syndrome at 6 months of 0.71 (95% CI, 0.44-1.15) and at 18 months of 1.00 (95% CI, 0.63-1.58).

When the AO and CLT groups were combined, change in aerobic fitness significantly affected the odds of having metabolic syndrome at follow-up. At 6 and 18 months, a 1-beat-per-minute reduction in stage 2 heart rate was associated with a 4% reduction in the odds for metabolic syndrome prevalence (OR, 0.96; 95% CI, 0.94-0.98).

Weight loss was a significant mediator of lower metabolic syndrome prevalence at 6 and 18 months (Table 2). At 6 months, an increase in aerobic fitness was associated with lower odds of all cardiometabolic risk factors (Table 3). With each beat-per-minute decrease in stage 2 heart rate, the odds of having a given component ranged from 2% lower for high blood pressure to 7% lower for high waist circumference and high fasting glucose. We found the results to be attenuated at 18 months, and improved aerobic fitness was no longer associated with lower odds for high triglycerides. When controlling for weight change, aerobic fitness change was no longer associated with reduced odds of high blood pressure, high triglycerides, or high waist circumference at 6 months. At 18 months, only the odds of high blood pressure remained significant after weight change was included in models.

## Discussion

In our study population, the absolute prevalence of metabolic syndrome decreased from baseline to 6 months by 13 percentage points and from baseline to 18 months by 15 percentage points in the AO control group. In the CLT group, prevalence decreased by 19 percentage points and 17 percentage points at 6 and 18 months, respectively. Stewart et al reported that after a 6-month exercise trial, the prevalence of metabolic syndrome decreased by approximately 18 percentage points in the exercise group and 8 percentage points in controls (17). The prevalence decline we found in the AO group may be explained because AO was not a "true" control group. These participants also improved their fitness and lost weight. We previously reported a mean 1.1 kg reduction in weight in the AO group at 6 months (19).

We hypothesized that change in aerobic fitness from CLT would result in lower prevalence of metabolic syndrome relative to AO. Our findings did not support this hypothesis. The modest improvement in fitness in AO, while significantly less than in the intervention groups (19), may have been sufficient to attenuate differences between AO and CLT. However, our results were similar to those of Finucane et al, who did not observe a reduced metabolic syndrome score after a 12-week intervention conducted among older adults (18). Given the high prevalence of Americans with metabolic syndrome (6), more studies are needed to identify appropriate lifestyle interventions.

At 6 and 18 months, aerobic fitness predicted metabolic syndrome prevalence, independent of intervention or control group status. Our results agree with previous cross-sectional (29-31) and longitudinal studies (9-13). Of note, we found this association to be mediated by change in weight. Using prospective cohort designs, some studies found physical activity or fitness to predict metabolic syndrome incidence independent of baseline weight status (9,12,13), although another did not (11). Carnethon et al, in a study predicting metabolic syndrome incidence over 6 years, found that when change in weight and change in physical activity were included in multivariable models, only change in weight predicted metabolic syndrome incidence (10). In contrast, Rhéaume and colleagues found that change in fitness and change in abdominal fat was associated with change in a metabolic syndrome score (32). We found change in fitness and change in weight to be only moderately correlated (r = .40), so collinearity cannot completely explain our results and those of Carnethon and colleagues. Our analyses examining mediation suggest that weight change may drive the association we and others observed between fitness and metabolic syndrome. An intervention specifically designed to test the effects of improved fitness on metabolic syndrome in which some participants are randomized to a weight-loss program is needed.

Of the 5 risk factors included in the metabolic syndrome, all were significantly affected by an increase in aerobic fitness at 6 months, and all but high triglycerides at 18 months. These results are similar to those of Mathieu and colleagues, who reported that a physical activity and aerobic fitness intervention significantly improved blood pressure, waist circumference, and HDL cholesterol in participants with metabolic syndrome (33). Others found that all the metabolic syndrome risk factors were reduced because of an increase in fitness level after 3 years, although waist circumference was not measured (34). These studies did not control for change in weight, and we found that after including weight change as a covariate, most significant associations were lost. Aerobic fitness improvement does appear to benefit the metabolic syndrome components low HDL cholesterol and high fasting glucose.

This study had several limitations. PREMIER was not designed to examine change in metabolic syndrome prevalence and did not recruit participants who had metabolic syndrome. There may not have been sufficient distinction between AO and CLT with respect to change in fitness. The AO group, our control, received information about physical activity, weight loss, and dietary changes during 2 individual sessions with an interventionist. Participants in this group increased their fitness and, importantly, also lost weight. All participants were highly motivated and willing to participate in all aspects of the intervention and may not be comparable to the general population. Furthermore, our measurement of aerobic fitness may have introduced error. Stage 2 heart rate was used as a predictor of fitness. Although heart rate and oxygen uptake are highly correlated, a maximal oxygen uptake test was not conducted. We promoted physical activity rather than fitness in the intervention and results may be different from a fitness-specific intervention. We lost 146 participants because of missing follow-up fitness tests, potentially biasing the results. Finally, we implemented the study approximately 10 years ago. Today, we might focus on different interventions and measurements. Nevertheless, our results are valid.

Our findings suggest there is an association between increased aerobic fitness and reduced prevalence of metabolic syndrome for up to 18 months. These results appear to be mediated through concomitant weight change. Our results support the implementation of health promotion programs aimed at increasing physical activity, fitness, and weight change to reduce the prevalence of metabolic syndrome, especially in a population with high blood pressure.

## Figures and Tables

**Table 1.** Baseline Characteristics by Treatment Group of PREMIER Trial, Louisiana, Maryland, Oregon, North Carolina, 1998-2004

**Characteristic**	Advice-Only Group, %^a^ (n = 271)	Combined Lifestyle Treatment Group,^b^ %^a^ (n = 533)
Age, mean (SD), y	49.5 (8.9)	50.2 (8.9)
Female	63.5	61.5
**Race/ethnicity**		
African American	36.9	33.0
Non-African American	63.1	67.0
**Annual household income, $**		
<30,000	14.0	8.4
30,000-60,000	30.3	32.3
>60,000	51.7	55.9
Unknown (no answer)	4.1	3.4
**Education**		
High school graduate or less	10.7	8.4
Some college	60.5	58.2
Some graduate school	28.8	33.4
**Current cigarette smokers**	5.0	5.0
**BMI, mean (SD), kg/m^2^**	32.9 (5.6)	33.2 (5.9)
**Weight classification**		
Normal weight (BMI <25.0)	4.8	4.5
Overweight (BMI 25.0-29.9)	27.7	29.8
Obese (BMI >30.0)	66.8	64.7
**Stage 2 heart rate, mean (SD), beats/min^c^**	130.2 (14.5)	130.1 (14.4)
**Metabolic syndrome prevalence**	47.2	49.9
**Metabolic syndrome risk factors^d^**		
High waist circumference	79.3	80.5
High triglycerides	31.7	34.3
Low HDL cholesterol	43.5	49.0
High blood pressure	74.5	77.9
High fasting glucose	9.6	14.1

Abbreviations: SD, standard deviation; BMI, body mass index; HDL, high-density lipoprotein.

a Values are percentages except where noted.

b Combination of the Established Guidelines group and the Established Guidelines plus Dietary Approaches to Stop Hypertension diet group from original PREMIER study (19).

c Determined from a submaximal treadmill test.

d Defined by National Cholesterol Education Panel-Adult Treatment Panel III guidelines as ≥3 of the following: high waist circumference, >88 cm for women or >102 cm for men; high triglycerides, >149 mg/dL; low HDL cholesterol, <50 mg/dL for women or <40 mg/dL for men; high blood pressure, >129/84 mm Hg or medication; high fasting glucose, >109 mg/dL (22).

**Table 2.** Prevalence of Metabolic Syndrome, by Change in Aerobic Fitness and Weight, at 6- and 18-Month Follow-Up, PREMIER Trial, Louisiana, Maryland, Oregon, North Carolina, 1998-2004

**Aerobic Fitness/Weight Change**	β (SE)^a^	*P* Value
**6 Months (n = 665)**		
**Step 1^b^**		
1 beat change in heart rate^c^	−0.04 (0.01)	<.001
**Step 2^d^**		
1 kg change in weight	0.08 (0.01)	<.001
**Step 3^e^**		
1 kg change in weight	0.08 (0.01)	<.001
1 beat change in heart rate^c^	−0.01 (0.01)	.32
**18 Months (n = 656)**		
**Step 1^b^**		
1 beat change in heart rate^c^	−0.04 (0.01)	<.001
**Step 2^d^**		
1 kg change in weight	0.05 (0.01)	<.001
**Step 3^e^**		
1 kg change in weight	0.04 (0.01)	<.001
1 beat change in heart rate^c^	−0.02 (0.01)	.13

Abbreviation: SE, standard error.

a Logistic regression model for combined intervention and control groups controlled for site, cohort, age, sex, race, baseline body weight, baseline fitness, baseline metabolic syndrome, urinary sodium and daily intakes of alcohol, dairy, fruits, vegetables, total fat, and fat servings.

b Step 1: Change in aerobic fitness regressed on metabolic syndrome prevalence.

c Aerobic fitness defined as 1 beat/min lower heart rate at stage 2 of VO_2_ max treadmill test.

d Step 2: Change in weight regressed on metabolic syndrome prevalence.

e Step 3: Change in aerobic fitness regressed simultaneously on change in weight and metabolic syndrome prevalence.

**Table 3.** Odds of Metabolic Syndrome Indicators, by Aerobic Fitness Change and Weight Change, at 6- and 18-Month Follow-Up, PREMIER Trial, Louisiana, Maryland, Oregon, North Carolina, 1998-2004

Model Parameter	Metabolic Syndrome Indicator^a^^,^ b									
	High BP, OR (95% CI)	*P*	High Triglycerides, OR (95% CI)	*P*	High WC, OR (95% CI)	*P*	Low HDL-C, OR (95% CI)	*P*	High Fasting Glucose, OR (95% CI)	*P*
**6 Months (n = 665)**										
Aerobic fitness change^c^ without weight change in model	0.98 (0.96-0.99)	.01	0.97 (0.94-0.99)	.005	0.93 (0.90-0.97)	<.001	0.94 (0.91-0.97)	<.001	0.93 (0.90-0.97)	<.001
Aerobic fitness change^c^ with weight change in model	0.99 (0.97-1.01)	.47	0.99 (0.97-1.02)	.63	0.97 (0.94-1.01)	.16	0.95 (0.91-0.98)	.003	0.94 (0.91-0.98)	.005
**18 Months (n = 656)**										
Aerobic fitness change^c^ without weight change in model	0.97 (0.95-0.98)	.001	0.99 (0.96-1.01)	.14	0.95 (0.92-0.98)	.001	0.97 (0.94-1.00)	.03	0.97 (0.94-1.00)	.009
Aerobic fitness change^c^ with weight change in model	0.98 (0.96-1.00)	.05	1.00 (0.98-1.03)	.85	1.00 (0.96-1.03)	.78	0.98 (0.95-1.01)	.24	0.97 (0.94-1.01)	.16

Abbreviations: BP, blood pressure; OR, odds ratio; CI, confidence interval; WC, waist circumference; HDL-C, high-density lipoprotein cholesterol.

a Defined by National Cholesterol Education Panel-Adult Treatment Panel III guidelines as the presence of ≥3 of the following risk factors: high WC, >88 cm for women or >102 cm for men; high triglycerides, >149 mg/dL; low HDL-C, <50 mg/dL for women or <40 mg/dL for men; high BP, >129/84 mm Hg or medication; high fasting glucose, >109 mg/dL (22).

b Logistic regression model for combined intervention and control groups of original PREMIER study (19) controlled for site, cohort, age, sex, race, baseline body weight, baseline fitness, baseline metabolic syndrome, urinary sodium, and daily intakes of alcohol, dairy, fruits, vegetables, total fat, and fat servings.

c Aerobic fitness defined as 1 beat/min lower heart rate at stage 2 of submaximal treadmill test.
